# Public Health Offenders

**Published:** 1920-02-07

**Authors:** 


### PUBLIC HEALTH OFFENDERS.

There was something more than satire in Samuel Butler's "Erewhonian" law which made ill-health an offence. Like most satire, it was merely the overstatement of a truth. For it is almost a platitude to say that with the reasonable exercise of the simple laws of health much sickness could easily be avoided. It is a reflection on our national sanity that the Ministry of Health should consider it necessary—and rightly so—to issue a set of hints on how best to minimise the chances of infection from influenza germs. They are a few of the fundamental laws of good health, with the single exception of experimental inoculation. Be properly clothed, have the homes and offices adequately ventilated, eat regularly, avoid over-indulgence in liquor, keep away as far as possible from overcrowded meeting-places, and eschew all unnecessary travelling in 'buses and on railways. But there will doubtless be people who, while disobeying these advices—some of them, it is true, compelled to be ignored by force of circumstances—will faithfully gargle and rush to be inoculated. Did not Naaman hesitate before acting upon the simple injunction to dip himself seven times in the Jordan in order to be cured of leprosy?

But people, if they do not bother about a duty to themselves, have at least a duty to others in an effort to prevent the spread of disease. They cannot avoid travelling in the scandalous conditions of the present time, and overcrowding in houses cannot nowadays be made the responsibility of the individuals. Anyone who has to take part in the appalling daily scramble for some footing in train or 'bus may be excused bitterness and irritation at being warned of the danger. They are compelled to offend by becoming unduly fatigued, by risking chills through long waits, by breathing bad air, and by regular overcrowding. But it is a disgusting and obvious fact that so many ignore the warnings against spitting, or sneezing and coughing in a neighbour's face. It is a pity that by-laws against spitting are not rigorously enforced. If people cannot behave with decency towards their fellows, they ought at least to be shown that they will not be allowed to continue their course with impunity. There seems, however, nothing to be done with the fellow who puffs his tobacco smoke in your face, and coughs at you without the faintest effort at protection. He does it with the same sublime oblivion as the man who wears brown boots with morning dress, but while the latter merely amuses us for his ignorance of "what is done," the former is nauseating. Things are probably better than they were, but the Ministry of Health is to be commended for its understanding in emphasising the simple virtues. They need emphasis on lines similar to the "safety first" campaign, though the issue is not quite so plain. No man is fool enough to advocate disregard of safety precautions, but there are cranks ready to preach the advantages of uncleanliness and like absurdities.

